# Bone defect map of the true acetabulum in hip dysplasia (Crowe type II and III) based on three-dimensional image reconstruction analysis

**DOI:** 10.1038/s41598-021-02448-z

**Published:** 2021-11-25

**Authors:** Xinggui Wen, Jianlin Zuo, Tong Liu, Zhongli Gao, Jianlin Xiao

**Affiliations:** 1 Department of Hand Surgery, China-Japan Union Hospital of Jilin University, 126 Xiantai Avenue, Changchun, 130033 Jilin China; 2 Department of Orthopedics, China-Japan Union Hospital of Jilin University, 126 Xiantai Avenue, Changchun, 130033 Jilin China

**Keywords:** Preclinical research, Bone development, Bone

## Abstract

The high hip center technique (HHC) is considered to be feasible for acetabular reconstruction in patients with DDH, but there is little in-depth study of its specific impact on Crowe type II and III DDH. The purpose of this study was to simultaneously analyze the effect of HHC on bone coverage of the cup (CC) in the acetabular reconstruction of type II and III DDH patients and to propose a map of acetabular bone defects from the perspective of the cup. Forty-nine hip CT data of 39 patients with DDH (Crowe type II and III) were collected to simulate acetabular reconstruction by cup models of different sizes (diameter 38mm–50 mm, 2 mm increment) with the HHC technique. The frequency distribution was plotted by overlapping the portions of the 44 mm cups that were not covered by the host bone. The mean CC of cups with sizes of 38 mm, 40 mm, 42 mm, 44 mm, 46 mm, 48 mm, and 50 mm at the true acetabula were 77.85%, 76.71%, 75.73%, 74.56%, 73.68%, 72.51%, and 71.75%, respectively, and the maximum CC increments were 21.24%, 21.58%, 20.86%, 20.04%, 18.62%, 17.18%, and 15.42% (*P* < 0.001), respectively, after the cups were elevated from the true acetabula. The bone defect map shows that 95% of type II and III DDH acetabula had posterosuperior bone defects, and approximately 60% were located outside the force line of the hip joint. Acetabular cups can meet a CC of more than 70% at the true acetabulum, and approximately 60% of Crowe type II and III DDH patients can obtain satisfactory CC at the true acetabulum by using a 44-mm cup without additional operations.

## Introduction

The best approach for placement of the acetabular component in total hip arthroplasty (THA) remains controversial for patients with developmental dysplasia of the hip (DDH), especially for those with Crowe type II and type III. When the acetabular cup is placed at the level of the true acetabulum, the bone defect caused by acetabular dysplasia may result in poor cup coverage (CC) in THA^1,2^, leading to unsatisfactory initial stability and a low long-term survival rate^3–5^. To achieve adequate CC, several studies^6–8^ have recommended bone grafting, the medial protrusion technique, and the use of extra-small cups at the true acetabulum during acetabular reconstruction. However, it remains disputable whether these techniques are helpful for reconstructing the acetabulum in DDH patients because these techniques have various complications and uncertain long-term survival rates^9–11^.

The high hip center (HHC) technique, placing the acetabular cup above the true acetabulum during acetabular reconstruction, is suitable for DDH patients because it can make full use of the bone above the true acetabulum to ensure adequate host bone coverage of the cup^12,13^. In addition, the HHC technique is associated with some advantages, such as reduced additional surgery and shortened operation time^12^. Based on three-dimensional (3D) reconstruction technology, several studies have shown that the HHC technique can greatly increase the bone coverage of the cup^14–16^, which may result in significant increases in the initial stability of the cup, bone ingrowth, and long-term survival. Our previous study on the morphological features of the true acetabulum in DDH patients showed that the bone stock at the true acetabulum of Crowe type I and Crowe type IV patients was sufficient to support acetabular reconstruction, but for type II and type III patients, additional surgery may be required for in situ acetabular reconstruction^17^. However, these studies did not conduct in-depth research on acetabular reconstruction evaluation of type II and type III DDH.

In 2009, Armitage et al.^18^ analyzed the distribution of fracture lines with 3D computed tomography (CT) of the scapula and proposed the concept of fracture maps. In recent years, many studies have reported analyses of different fracture maps^19–21^. To the best of the authors’ knowledge, no study has introduced the concept of fracture maps into the field of joint replacement and related research and investigated the distribution of acetabular bone defects in patients with type II and type III DDH from the perspective of uncovered acetabular cups. If these two ideas are combined to create a bone defect distribution map, the distribution of bone defects of the true acetabula in DDH patients will be intuitive and clear.

Since a 44-mm cup is the smallest one available for ceramic-ceramic cementless prostheses, some studies regard a 44 mm cup as a standard cup^15,16^. In the present study, 44-mm cup models with fixed positions were used to evaluate the bone defects of the true acetabula and map the frequency distribution in patients with Crowe type II and type III DDH to make the results of the evaluation unified and standardized. Therefore, the map can be used to guide surgeons to reconstruct the acetabula in patients with Crowe type II and type III DDH in clinical work.

## Research methodology

This study was based on three-dimensional reconstruction images, patients from the China-Japan Union Hospital were selected as research objects, and their pelvic CT data were collected for study.

### Patients

This study was approved by the Institutional Review Board, Ethics Committee of the China-Japan Union Hospital (IRB No: 2016ks001). All patients signed informed consent before X-ray and CT examination. All operations and experiments were carried out in strict accordance with the relevant regulations and guidelines. The present study included 49 hips of 39 patients (3 males and 36 females) who underwent hip X-ray and CT examination in our hospital. According to the Crowe classification^1^, 28 hips were classified as type II, and 21 hips were type III. None of the patients had hip diseases, including avascular necrosis of the femoral head and infection or a history of hip surgery, such as pelvic osteotomy, bone grafting, and osteotomy of the femur. The demographic data are shown in Table 1.

**Table 1:** Demographic characteristics of the patients.

Characteristic	Value
Number of patients (hips)	39 (49)
Gender (male/female)	3/36
Age (Years)^a^	48.92 ± 10.69
Height (cm)^a^	158.59 ± 5.78
Weight (kg)^a^	60.74 ± 9.65
BMI (body mass index, kg/m^2^)^a^	24.11 ± 3.38
Unilateral(left/right)	29 (12/17)
Bilateral	10
**Crowe type (number of hips)**	
II	28
III	21

^a^Values are expressed as means ± SD.

### CT scan and 3D reconstruction

CT scans of the entire pelvis were performed with a Toshiba Aquilion CT scanner (120 kVp, 320 mA, 512 × 512 matrix, and 0.5-mm slice thickness). The patients were placed in a supine position on the CT table with the patellae facing the ceiling, and the axis of the body coincided with the axis of the examination table. All CT data were saved in Digital Imaging and Communications in Medicine (DICOM) format, imported into Mimics 17.0 software (Materialise, Version 17.0.0.435, RUL: https://www.materialise.com/en/medical/mimics-innovation-suite/mimics) and used to reconstruct 3D images for each pelvis. The positions of the 3D reconstruction images were adjusted according to the anterior plane of the pelvis determined by the anterior superior iliac spine and pubic tubercle^17^.

### Simulating implantation of the acetabular cup model

**Figure 1:**
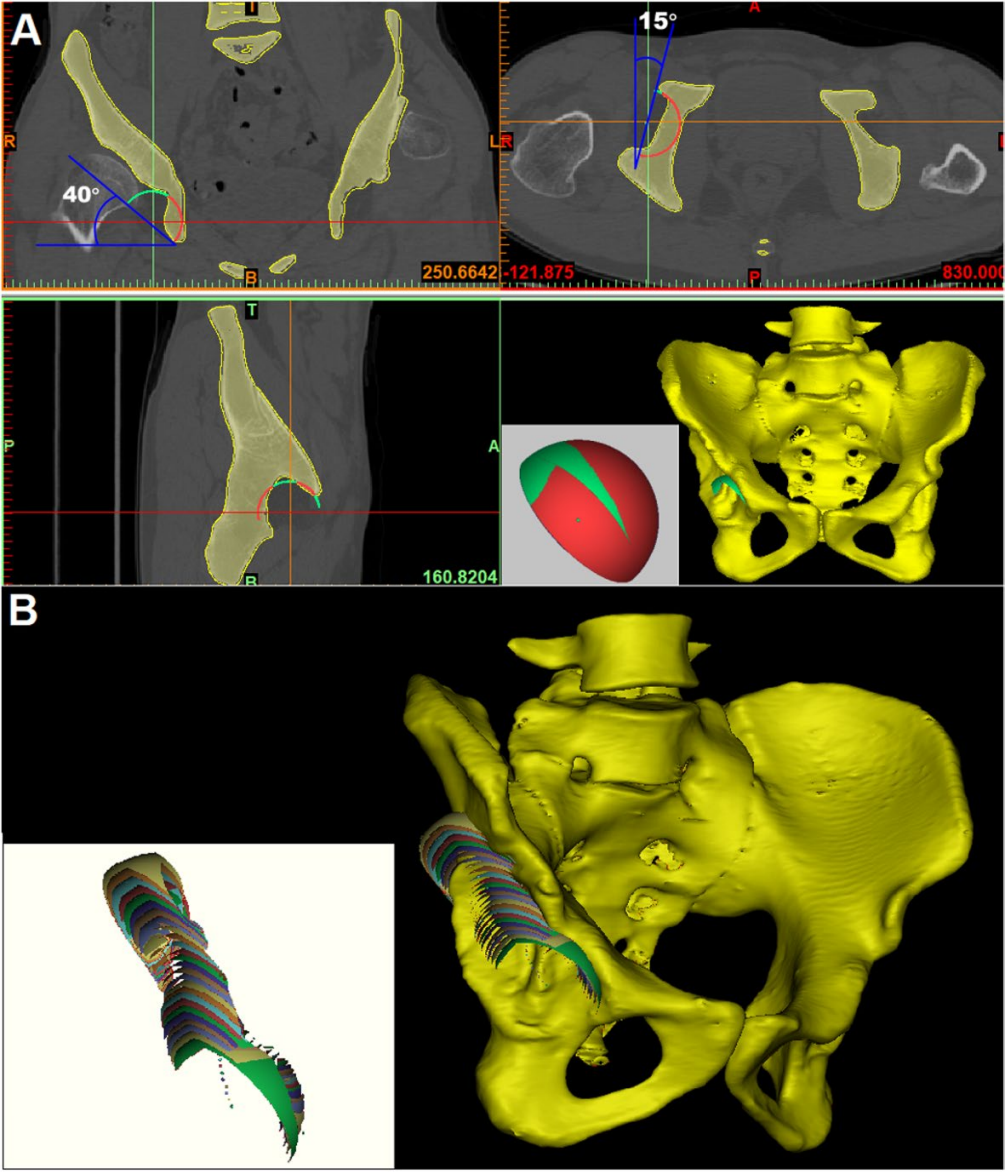
3D images of the pelvis reconstructed by Mimics software (Materialise, Version 17.0.0.435, RUL: https://www.materialise.com/en/medical/mimics-innovation-suite/mimics) using complete CT data of the pelvis. (**A**) The cup models were placed in the 3D reconstruction image of the pelvis to simulate acetabular reconstruction in THA. The position of the cup was adjusted to 40° of abduction and 15° of anteversion on the coronal and transverse sections. The total surface area of the cup (S_t_) can be read directly from the Mimics software. Boolean operation was used to obtain the uncovered area of the cup (S_u_), which is indicated in green; the covered area is indicated in red. (**B**) The uncovered portions of the cup with a diameter of 50 mm at different elevations are indicated in different colors.

We used a set of acetabular cup models created in our previous study^17^. The diameter of the cups ranged from 38 to 50 mm in 2-mm increments. All cups had a shell thickness of 0.1 mm. These models were imported into the Mimics software in stereolithography (STL) format, and the total surface area (S_t_) was available. The cups were implanted into the reconstructed 3D images of the pelvis using the Mimics software, and the method of simulating acetabular cup implantation in THA was as described in our previous study^17^. The position of all the cups was set at 40° abduction and 15° anteversion constantly (Fig. 1A).

### Measurement of the CC

To measure the CC, the cups were placed at the true acetabulum firstly^22^ and then elevated upward in 2‑mm increments to 50 mm (Fig. 1B). At each elevation, the cup models were adjusted slightly in coronal and transverse sections to achieve the maximum CC. After the cup models were placed in the 3D reconstruction image of the pelvis, the surface area of the uncovered portion (S_u_) was read by the Boolean operation function of Mimics. The CC of cups with different diameters (ranging from 38 to 50 mm) at each elevation was calculated by the following formula:

$$CC = \left( {S_{t} - S_{u} } \right)/S_{t} \times 100\%$$

### Mapping the uncovered portions of acetabular cups

The uncovered portions of all 44-mm cups at the true acetabula were imported into Magics 22.03 software (Materialise) in STL format to overlap onto a complete cup model. The uncovered portions of all 44-mm cups were superimposed to create a compilation of uncovered portions on a complete cup model serving as a representation of the exposed portion of the acetabular cups in THA. The overlap of all uncovered portions of the cups was used to build a distribution map of frequency based on the density of the uncovered portions of the cups, and then the distribution frequency was calculated.

### Analysis of the defect distribution

A vertical line was made through the rotation center of the cup, and then a plane perpendicular to the anterior plane of the pelvis was built based on this line. Therefore, the distribution frequency of the uncovered portion of the cups outside the gravity line of the hip joint could be calculated. The probability of undergoing additional surgery, such as bone grafting, in acetabular reconstruction at the true acetabulum in patients with Crowe type II and type III DDH could also be calculated.

### Statistical analysis

All data are expressed as the means ± standard deviation (SD). A paired t test was used to evaluate the differences between the CC at the true acetabulum and the maximum CC. Pearson's correlation coefficient was used to evaluate the correlation between CC and the elevated height of the cup. All statistical analyses were performed using SPSS 21.0 software (SPSS, Chicago, IL, USA). Statistical significance was defined as *P* < 0.05.

## Results

**Figure 2:**
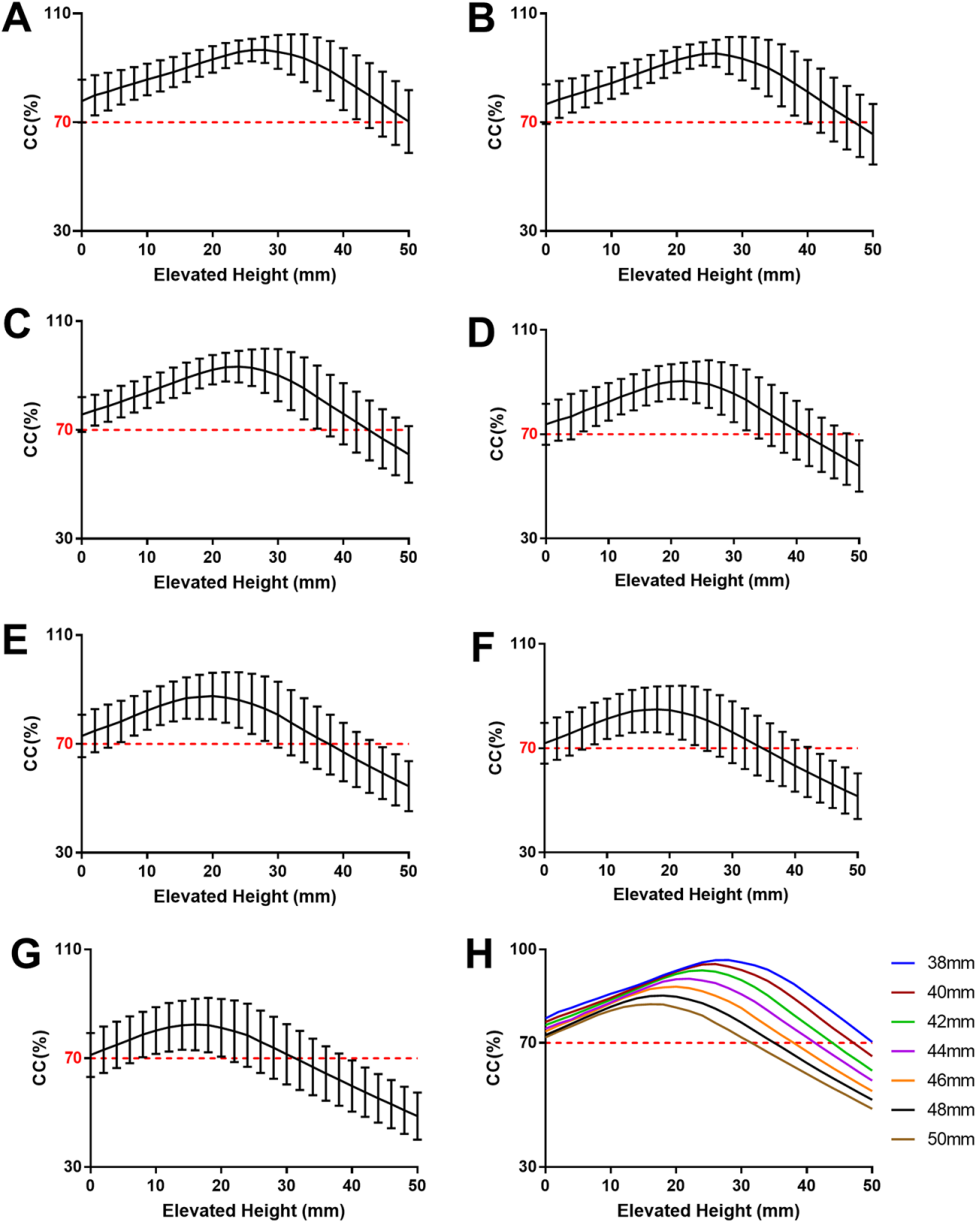
Changes in CC and the elevated height of the cup from the true acetabulum for cup sizes of 38 mm (**A**), 40 mm (**B**), 42 mm (**C**), 44 mm (**D**), 46 mm (**E**), 48 mm (**F**), and 50 mm (**G**). **H** shows the relationship between the CC and the elevated height of cups of different sizes. With increasing cup size, the elevated height of the cup decreased gradually when the maximum CC was reached.

The CC of all cups with different sizes at the true acetabulum was greater than 70%, and as the acetabular cups elevated from the true acetabulum, the CC gradually increased until reaching the maximum and then decreased gradually to below 70% (Fig. 2). When the CC reached the maximum and decreased to less than 70%, the corresponding elevated height of the cups decreased with increasing cup size. The CC of all cups at the true acetabulum was greater than 71%, all the cups reached a maximum CC greater than 87% after elevating from the true acetabulum, and the maximum CC was significantly different from that at the true acetabulum (Table 2). When the CC reached the mean maximum value of 93.96%, the cup was elevated to a mean height of 23.76 mm from the true acetabulum, and the CC increased by 19.28% (*P* < 0.001). The mean CC of 44-mm cups at the true acetabulum was 74.56%, and it reached a maximum of 94.61% after elevating 24.16 mm from the true acetabulum.

**Table 2:** The CC at the true acetabula, the maximum CC and the elevated height when CC meet the maximum.

Cup size (mm)	CC at true acetabulum (%)^a^	Maximum CC (%)^a^	Difference (%)^a^	Elevated height (mm)^a^
38	77.85 ± 7.88	99.09 ± 2.21	21.24 ± 8.27^b^	29.14 ± 5.85
40	76.71 ± 7.28	98.30 ± 3.07	21.58 ± 7.62^b^	27.84 ± 5.51
42	75.73 ± 6.40	96.59 ± 4.43	20.86 ± 6.94^b^	25.96 ± 5.77
44	74.56 ± 6.21	94.61 ± 5.67	20.04 ± 6.88^b^	24.16 ± 6.16
46	73.68 ± 6.27	92.30 ± 7.17	18.62 ± 6.54^b^	21.76 ± 5.83
48	72.51 ± 6.65	89.69 ± 8.51	17.18 ± 7.01^b^	19.59 ± 6.51
50	71.75 ± 7.23	87.17 ± 9.55	15.42 ± 7.07^b^	17.88 ± 7.12
Mean	74.69 ± 7.11	93.96 ± 7.52	19.28 ± 7.46^b^	23.76 ± 7.22

^a^Values are presented as mean ± SD.

^b^*P* < 0.0001, paired t test.

**Figure 3:**
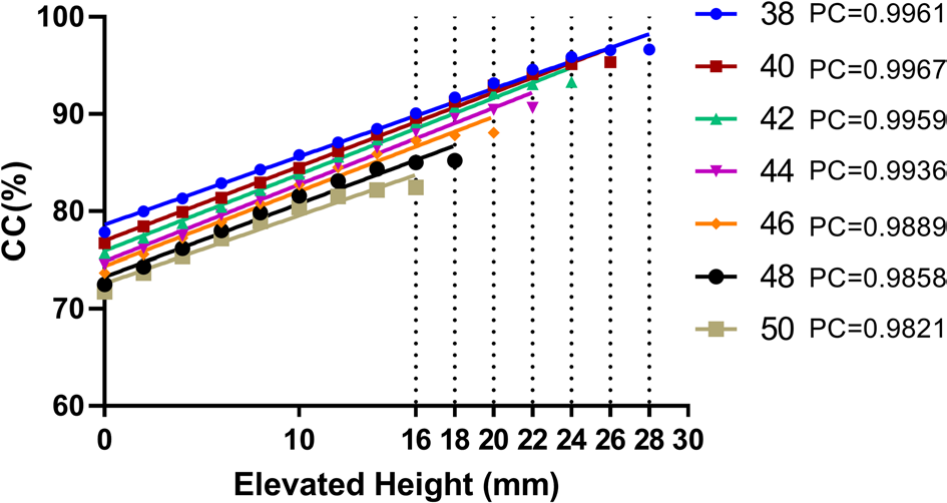
Relationship of the CC and elevated height of cups from the true acetabulum until the CC reached the maximum value. PC: Pearson correlation coefficient.

The CC values were positively correlated with elevated height for cups with sizes of 38 mm, 40 mm, 42 mm, 44 mm, 46 mm, 48 mm, and 50 mm, and the Pearson's correlation coefficients were 0.9961, 0.9967, 0.9959, 0.9936, 0.9889, 0.9858, and 0.9821, respectively, indicating that the correlation between CC and elevated height was almost linear in all cups (Fig. 3). The mean elevated height at which the CC reached the maximum decreased with increasing cup size.

**Figure 4:**
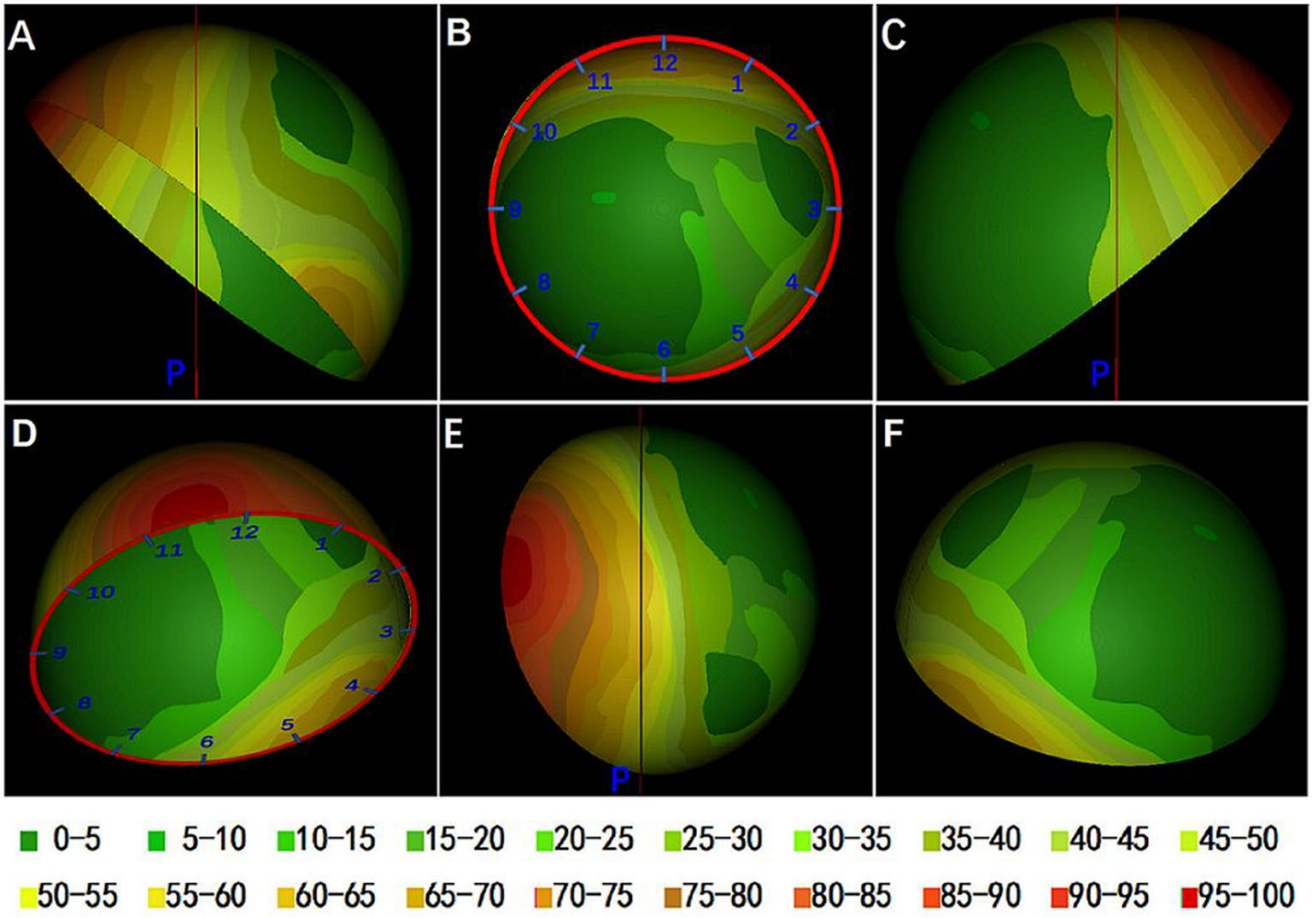
The distribution frequency of the uncovered portion on intact cup is shown in different colors. The result is expressed as a percentage with a total of 49. A clock pattern was drawn on the cup to show the location of the uncovered portion. A plane perpendicular to the anterior plane of the pelvis was built through the rotation center of the cup. (**A**) Front view of the pelvis. The red line is a vertical line through the rotation center of the mortar cup. (**B**) Front view of the cup. (**C**) Rear view of the cup. (**D**) Vertical view of the cup. p: Vertical plane through the rotation center of the cup.

The exposed portion of cups was mainly (> 60%) concentrated in the posterosuperior, and a small area located in the area between 11 and 12 o'clock on almost all the cups was not covered by the host bone, as shown in crimson (Fig. 4). Approximately 50–70% of the uncovered portions were concentrated in the anteroinferior of the cup, and surprisingly, approximately 10–25% of the uncovered portions were concentrated in the medial of the cup. The uncovered portions of several special cases accounted for the frequency of 5–10% concentrated in the posteromedial of the cup.

Over 60% of the uncovered portions of the cups were outside the gravity line of the hip, so it can be inferred that when the 44-mm cup is used to reconstruct the acetabulum at the true acetabulum for Crowe type II and type III DDH patients, approximately 40% of the uncovered portions of the cups are located within the gravity line of the hip, and these hips may require additional surgery to ensure the initial CC in THA.

## Discussion

According to previous studies^1^, for Crowe type II and type III DDH patients, it is difficult to achieve satisfactory CC when reconstructing the acetabulum due to shallow acetabulum and bone defects. Placing the cup in the bone stock above the true acetabulum with sufficient bone mass can increase the CC and ensure the initial stability of the acetabular cup^12–16^. Here, it was found that elevating cups of different sizes to 23.76 mm above the true acetabulum increased the CC by 19.28% during acetabular reconstruction for patients with Crowe type II and type III DDH, which is consistent with the findings of previous studies^13–16,23^. In addition, the mean elevated height identified in this study meets the safety range determined by Hirakawa et al.^24^, who recommended that the cup should be placed < 35 mm vertically from the interteardrop line.

Sufficient host bone coverage of the acetabular cup is required to achieve satisfactory long-term implant survival for DDH patients following THA. Many studies have demonstrated that the HHC technique is a feasible method for reconstructing the acetabulum at the level of the true acetabulum in DDH patients^14–16^. In this study, except for the 48-mm and 50-mm cups, the cups achieved a mean CC of greater than 90% after elevating from the true acetabulum. Many studies have shown that the HHC technique does not cause aseptic loosening of cups, and the outcomes after medium- and long-term follow-ups are satisfactory^13,25–27^. Therefore, we believe that the HHC technique is a valid method of reconstructing the acetabulum for DDH patients.

Few studies have reported an analysis of acetabular reconstruction for Crowe type II and type III DDH patients based on CT of the pelvis and acetabular cup prosthesis models. Komiyama^28^ used a cup with a diameter of 50 mm and positioned the cup with an inclination of 40° and an anteversion of 20° to simulate acetabular reconstruction in 32 patients with Crowe type II and type III DDH. The cup center-edge (Cup-CE) angle was used to evaluate the CC. The results showed that at the anatomical hip center, the mean Cup-CE was − 4.3 ± 11.8°, 13 hips (40.6%) satisfied Cup-CE ≥ 0°, and a Cup CE angle ≥ 0° was used as the cutoff value for the required bone coverage in their study, which was approximately equal to a bone coverage of the acetabular cup ≥ 60%. Xu et al.^16^ reported acetabular reconstruction for Crowe IV DDH patients with a 44-mm cup at a position of 40° abduction and 20° anteversion, and the simulated mean in situ 3D CC was 78.60% (67.67–92.51%). In 2018, Liu et al.^15^ evaluated CC at the true acetabulum for 20 Crowe type III DDH hips with a 44 mm cup model. The inclination was set to 45° constantly, and three anteversion groups with 0°, 5°, and 10° were set. The CC was 65.87% ± 7.82%, 67.77% ± 8.02%, and 68.98% ± 6.97%, respectively. To our knowledge, the present study is the first to evaluate CC at the true acetabulum for Crowe type II and III DDH patients based on 3D reconstruction images, and the results are different from previous views and studies; that is, using cups with diameters of 38 mm, 40 mm, 42 mm, 44 mm, 46 mm, 48 mm, and 50 mm to reconstruct the acetabulum, the cups can obtain a mean 3D bone coverage of 74.69% at the true acetabulum. According to the existing literature^29–31^, no additional operation is required to ensure the initial stability of cups during acetabular reconstruction if the CC is greater than 70%.

This study analyzed the CC of 44-mm cups at the true acetabulum and the distribution of uncovered portions. Many existing studies^14,17,32–34^ have described bone defects of the true acetabulum in DDH patients from the perspective of pelvic morphology, but few studies^15,16^ have analyzed the uncovered portion from the perspective of acetabular cups. Setting a proper position of the acetabular cup is the only action that can be taken during THA, so it is most important to carry out relevant research from the perspective of the acetabular cup. The present study is the first to analyze the uncovered portion of cups in THA and to draw a map of the exposed portion of cups that represents bone defects of the true acetabulum. From this map, the distribution and probability of the uncovered portion of the acetabular cup can be seen intuitively. The results show that 40% of the uncovered portions of cups are located inside the gravity line of the hip, and we believe that if the uncovered portion of the cup is located inside the gravity line of the hip joint, hearing forces on the acetabular cup may lead to early loosening^4^. Therefore, it can be inferred that approximately 60% of type II and type III DDH patients can obtain satisfactory CC without additional operations when reconstructing the acetabula at the true acetabulum with 44-mm cups. This proportion is not very large, but it is truly a surprising finding. That is, during acetabular reconstruction for type II and type III DDH patients, if careful preoperative design is carried out, in many patients, placing an acetabular cup at the true acetabulum can also yield good CC, and initial stability can be ensured.

On the other hand, from the results of this study, if the acetabular bone defect is too large and bone grafting is necessary, the bone defect distribution map can be used as a guide for orthopedic surgeons to determine the location of bone grafts during preoperative design. As mentioned above, previous studies^14,17^ analyzed acetabular bone defects with pelvic morphology, which was indeed a direct description of bone defects. However, due to the individual variability of acetabular morphology in different DDH patients, it is impossible to describe the distribution of acetabular bone defects in a unified way. In the present study, acetabular cup models with fixed size and position were used to evaluate the bone defects of the true acetabula, and then the frequency distribution was mapped to achieve unity and standardization of evaluation.

Our study has some limitations. First, the sample size was relatively small (n = 49 hips). Some patients underwent THA without CT data of the pelvis, thus making it impossible to perform 3D reconstruction and related analysis. Additionally, our surprising findings are based on 3D reconstruction images without clinical verification, and the long-term survival of acetabular prostheses is still unknown. Further study is being conducted to confirm our findings.

## Conclusion

Based on 3D reconstruction technology and surgical simulation technology, although the HHC technique can significantly improve acetabular cup coverage of the host bone in patients with Crowe type II and type III DDH, all cups can obtain a satisfactory CC (more than 70%) at the true acetabula during acetabular reconstruction. From the acetabular bone defect map (Fig. 4), approximately 60% of Crowe type II and type III DDH patients can obtain satisfactory CC of the host bone at the true acetabula by using a 44-mm cup without additional operations. With careful preoperative surgical planning and evaluation, orthopedic surgeons can perform acetabular reconstruction at the true acetabula for some Crowe type II and type III DDH patients without additional surgery.
